# Perceived family functioning and friendship quality: cross-sectional associations with physical activity and sedentary behaviours

**DOI:** 10.1186/s12966-015-0180-x

**Published:** 2015-02-21

**Authors:** Andrew J Atkin, Kirsten Corder, Ian Goodyer, Diane Bamber, Ulf Ekelund, Soren Brage, Valerie Dunn, Esther MF van Sluijs

**Affiliations:** MRC Epidemiology Unit & UKCRC Centre for Diet and Activity Research (CEDAR), University of Cambridge School of Clinical Medicine, Cambridge Biomedical Campus, Box 285, Cambridge, CB2 0QQ UK; Developmental Psychiatry Section, Department of Psychiatry, University of Cambridge, Cambridge, UK; Department of Sport Medicine, Norwegian School of Sports Sciences, Oslo, Norway

**Keywords:** Adolescents, Family, Peers, Physical activity, Sedentary behaviour

## Abstract

**Background:**

This study examined the association of adolescent-reported family functioning and friendship quality with objectively-measured moderate to vigorous physical activity (MVPA), sedentary time, and self-reported sedentary behaviours.

**Methods:**

Data are from the ROOTS study. MVPA and sedentary time were assessed using combined movement and heart rate sensing. Time spent TV viewing, using the internet, playing video games, doing homework and reading for pleasure was self-reported. Data on objectively-measured and self-reported outcomes for weekdays was available for 738 (age 14.5y, 55.7% female) and 800 (56.3% female) participants, respectively. Adolescents perceived family functioning and friendship quality (Two subscales: ‘Good friendship qualities’, ‘Friendship difficulties’) was assessed by questionnaire. Analyses were conducted using multi-level linear or logistic regression.

**Results:**

Adolescents reporting better family functioning accumulated more MVPA on weekdays (beta; 95% confidence interval: 0.57; 0.17,0.98). Higher scores on the good friendship qualities subscale was associated with greater MVPA throughout the week (weekdays: 1.13; 0.62,1.65, weekend: 0.56; 0.09,1.02) and lower sedentary time on weekdays (−10.34; −17.03,-3.66). Boys from better functioning families were less likely to report playing video games at the weekend (OR; 95% confidence interval: 0.73; 0.57,0.93) or reading for pleasure (weekday: 0.73; 0.56,0.96 weekend: 0.75; 0.58,0.96). Boys who attained higher scores on the good friendship qualities scale were less likely to play video games at the weekend (0.61; 0.44,0.86) or report high homework on weekdays (0.54; 0.31,0.94). A higher score for good friendship qualities was associated with lower odds of girls playing video games during the week (0.76; 0.58,1.00) or reading for pleasure at the weekend (0.61; 0.42,0.88). Girls that reported fewer friendship difficulties had lower odds of high TV viewing (0.76; 0.62,0.93) or playing video games (0.71; 0.52,0.97) at the weekend, and lower odds of reading for pleasure (0.63; 0.49,0.81) or reporting high homework on weekdays (0.70; 0.52,0.95).

**Discussion:**

Family functioning and friendship quality exhibit a complex pattern of association with physical activity and sedentary behaviour that varies by sex and day of the week. Findings highlight the potential value of targeting interpersonal aspects of the family and friendships as an adjunct to behaviour change interventions.

## Introduction

During childhood and adolescence, a physically active lifestyle may benefit metabolic and bone health, promote psychological well-being and protect against obesity [1,2]. However, a substantial proportion of young people fail to accumulate the recommended 60 minutes per day of moderate to vigorous intensity physical activity (MVPA) [3,4]. Moreover, levels of activity decline during the transition from childhood to adolescence, perhaps beginning as early as 10 years of age [5,6]. Accordingly, adolescents are a key population group for physical activity promotion; effective programmes implemented at this age may benefit health into adulthood. A growing body of research indicates that sedentary behaviours, such as TV viewing or travelling by motorised transport, may be detrimental to physical and psychological health, independently of participation in physical activity [7]. At present, however, longitudinal and experimental evidence linking sedentary behaviour with metabolic and mental health outcomes in young people is limited and it remains unclear whether some sedentary behaviours may be more harmful than others. Indeed, activities such as reading or homework, which are typically sedentary, serve important developmental and academic purposes, indicating the need for a balanced approach to the sedentary behaviour discourse and careful targeting of intervention programmes. In recognition of the potential public health burden of sedentary behaviour, guidelines have been established recommending that prolonged periods of sedentary behaviour should be limited [4].

The family is the primary unit of socialisation during childhood, and is central in shaping engagement in health behaviours, including physical activity [8-10]. To date, much of the research on familial influences on adolescent physical activity and sedentary behaviour has focused on specific parental or sibling factors, such as role modelling, parenting styles, rules or restrictions [10,11]. However, Family Systems Theory posits that the interactions within a family are reciprocal, such that each family member influences and is influenced by other family members [9,12]. Therefore, it is valuable to examine how broader characteristics of the family unit, such as communication and empathy, shape adolescents’ health behaviours. Family functioning refers to structural and organisational properties within a household, such as role behaviours and task organisation, and the nature of interpersonal relationships between family members, including communication, decision-making and problem solving. Poor family functioning has been associated with depressive symptomology [13], impaired academic performance [14] and disordered eating in adolescents [15], but few studies have examined its association with physical activity or sedentary behaviour. A recent analysis of US data found that participants indicating higher levels of family functioning reported greater physical activity (boys only) and lower screen-based sedentary behaviour (boys and girls) [16]. However, this study relied on self-report methods to assess physical activity and examined only a limited number of sedentary behaviours.

Alongside the family, friends and peers are a key influence on adolescents’ health behaviours [17,18]. Under certain circumstances, peers may exert a greater influence on health behaviours than parents [19]. Previous work in our group has shown that adolescents would prefer to be active with friends rather than parents or other family members [20]. It has been proposed that friendships may influence health behaviour through three mechanisms: social facilitation, role modelling and impression management [17]. Within the social facilitation domain, there is evidence that the qualitative nature of adolescents’ peer experiences may influence activity levels [17]. For example, simulated exclusion from a peer group was associated with lower levels of activity and greater sedentary behaviour in 11-year old children [21]. Taken together, these findings suggest that interventions aimed at improving peer relations and increasing friendship quality may be a route to promoting physical activity. However, previous studies in this area have predominantly used self-reports of adolescent activity and there is a lack of research into the influence of friendship quality on sedentary behaviours.

The aim of the current study was to examine the independent association of adolescent perceived family functioning and friendship quality with objectively measured physical activity, sedentary time, and self-reported sedentary behaviours in a large sample of early adolescents from the United Kingdom (UK). We hypothesised that participants indicating better family functioning and friendship quality would have higher levels of MVPA and lower sedentary behaviour and overall sedentary time.

## Methods

### Participants

Data are from ROOTS, a prospective cohort established primarily to determine the relative longitudinal contributions of genetic, physiological, psychological and social variables to well-being and the emergence of mental health problems during adolescence [22]. The ROOTS study was approved by the Cambridge Research Ethics Committee and sponsored by the Cambridge and Peterborough NHS Foundation Trust. Twenty-seven secondary schools located in the counties of Cambridgeshire and Suffolk (UK) were invited to participate and 18 agreed. Study information, invitation letters and parent and child consent forms were mailed to parents via schools. Of the 1238 eligible participants (those aged between 14 and 14 years 11 months during the allotted two-week interview period in each school) that were invited into the study, parent and child consents for the physical activity assessments were obtained from 998 participants (80.6% of eligible), of which 931 (93.3% of consenting) attended a testing session at school. All self-report data were collected between April 2005 and November 2006. Body composition and physical activity assessments were conducted approximately six months later, from November 2005 to July 2007.

### Family functioning and friendship quality

Family functioning was reported by adolescents using the 12-item general functioning subscale of the McMaster Family Assessment Device (FAD) [23,24] The general functioning scale provides a brief overarching measure of perceived family functioning. Example items: ‘Planning activities is difficult because we misunderstand each other’; ‘Individuals are accepted for what they are’. Response options were ‘strongly agree’, ‘agree’, ‘disagree’, and ‘strongly disagree’. Confirmatory factor analysis indicated adequate fit of a single factor model (Chi-square p < 0.001; Comparative fit index (CFI) = 0.92, Tucker-Lewis fit index (TLI) = 0.97, Root mean square error of approximation (RMSEA) = 0.11) and Cronbach’s alpha across the 12 items was 0.88. Factor scores from the confirmatory factor analysis model were derived by Mplus for use in the analysis, with higher scores indicating better perceived family functioning.

Friendship quality was rated by participants using an eight item questionnaire, which assesses the availability, adequacy and intimacy of current friendships [25]. The questionnaire includes items related to number of friends, frequency of seeing friends, confiding in friends and episodes of teasing. Example items: ‘Are you happy with the number of friends you’ve got at the moment?’; ‘Do your friends ever laugh at you or tease you in a hurtful way?’ Four to six response options were provided for each item. In factor analytic models, two underlying constructs were identified: 1) good friendship qualities, 2) friendship difficulties. Fit indices for the two factor model were adequate (Chi-square p < 0.001; CFI = 0.97, TLI = 0.93, RMSEA = 0.09). Factor scores for each construct were derived by Mplus for use in the analysis, with higher scores indicating better perceived friendship quality (i.e. more good friendship qualities / fewer friendship difficulties). There was a moderate positive correlation between scores on the two friendship constructs (rho = 0.6).

### Physical activity and sedentary behaviour

Physical activity and sedentary time were assessed objectively using combined heart rate and movement sensing (Actiheart, CamNtech Ltd, Papworth, UK) [26] Following an eight-minute step test for individual calibration of the heart rate–activity intensity relationship [27], the device was set-up to collect data in 30-second epochs and attached to the torso at the level of the sternum apex [28]. Volunteers were instructed to wear the monitor continuously, including during sleep and water-based activities, for the remainder of the testing day and then for four consecutive days, including two weekend days. Heart rate data were pre-processed [29], individually calibrated [27], and combined with acceleration in a branched equation framework [30] to estimate activity intensity time-series; these compare favourably with respiratory gas analysis measures of energy expenditure in laboratory evaluations [30,31]. Self-reported sleep times were overlaid on objective data and verified by visual inspection of plotted data to classify all time points as either awake or asleep. Prolonged periods of zero acceleration, accompanied by non-physiological heart rate data were classified as non-wear, which was taken into account when summarising time-series into physical activity outcome variables in order to minimise diurnal information bias. For inclusion in the analysis, monitor wear-time of ≥5 hours between both 9 am – 3 pm *and* 3 pm – 9 pm was required (minimum 10 hours total wear time). Data were derived initially at day-level, and subsequently summarised for weekdays and weekend days separately. One day of valid data was required for inclusion in weekday or weekend day analyses respectively. Moderate to vigorous intensity physical activity was defined as activity occurring at an intensity greater than four times energy expenditure at rest (4 METs). Sedentary time was defined as non-sleep activity occurring at less than 1.5 METs intensity [32].

Separately for week and weekend days, adolescents freely reported usual time spent per day in each of the following sedentary behaviours: watching TV (inc. video/DVD), using the internet, playing video games, doing homework, and reading for pleasure.

### Covariates

Participants self-reported their age and sex. Height (Leicester height measures; Chasmors Ltd, Leicester, UK) and weight (TBF-300A, Tanita, Tokyo, Japan) were measured by trained research assistants during school testing sessions. Body mass index (BMI) was calculated as weight (kg) / height^2^ (m). Neighbourhood-level socioeconomic position (SEP) was assessed using the ACORN (A Classification of Residential Neighbourhoods) index. ACORN categorizes UK post codes into five categories using 125 demographic and 287 lifestyle variables [33]. Categories were collapsed to represent high (categories 1/2), middle, and low (categories 4/5) SEP. Ethnicity was reported by parents. Due to limited heterogeneity, ethnicity was collapsed into white and non-white categories. Sexual maturity was categorised as early, middle or late, based upon self-assessed secondary sexual characteristics [34]. Current depressive symptomology was assessed using the Mood and Feelings Questionnaire (MFQ), a 33-item instrument with established validity for assessment of unipolar depression in adolescents [35]. Respondents rated their symptoms during the previous two weeks on a three-point scale (mostly/sometimes/never). A sum score was calculated, with higher scores indicating greater risk for depression.

### Statistical analysis

Factor analysis of the family functioning and friendship quality questionnaires was conducted in Mplus (Muthén & Muthén, 1998–2012). All other analyses were conducted in Stata (StataSE 12, College Station, TX). Characteristics of participants with missing data (relative to the analytical sample) and differences between boys and girls were assessed using Student’s t tests or *Χ*^2^ tests. To correct for skewness, objectively measured MVPA and sedentary time were Box-Cox transformed prior to analysis. All self-reported sedentary behaviours were highly skewed and distributions could not be normalised through transformation. TV viewing, internet use, and homework variables were dichotomised by median split (low coded 0; high coded 1). Due to the large number of zero values, playing video games and reading for pleasure were dichotomised as ‘none’ (coded 0) versus ‘some’ (coded 1). Multi-level linear or logistic regression models were used to examine the association of family functioning and friendship quality with physical activity and sedentary behaviour outcomes. Models were adjusted for school-level clustering, age, sex, BMI, ethnicity, sexual maturity, SEP, MFQ and included mutual adjustment for family functioning and friendship quality. In preliminary analyses, we examined effect modification by sex. School-level intra-class correlations ranged from 0.01-0.05 for objectively measured outcomes. No evidence of interaction was identified for either exposure with objectively measured outcomes. Significant interactions (P < 0.1) with sex were observed for all of the self-reported sedentary behaviours, either for weekdays or weekend days, with the exception of TV viewing. Therefore, analyses of objective outcomes were conducted for the entire sample, whilst those for self-reported outcomes were conducted separately for boys and girls.

## Results

Data on objectively measured outcomes was available for 738 and 677 participants on weekdays and weekend days respectively. Weekday and weekend data for self-reported outcomes was provided by 800 and 794 participants respectively. Compared to the sample providing valid objective physical activity data for either weekday or weekend analyses, participants with missing data had higher BMI (p = <0.01) but were no different in terms of age, sex or SEP. No demographic or anthropometric differences were observed between those with or without self-reported outcome data. Participant characteristics are presented in Table 1.

**Table 1:** **Participant characteristics (Data are median (IQR) unless stated otherwise)**

	**All**	**Girls**	**Boys**	***P***
**Gender, n (%)**	738	411 (55.7)	327 (44.3)	
**Age, y, mean (SD)**	14.5 (0.5)	14.5 (0.5)	14.5 (0.5)	0.19
**BMI, mean (SD)**	20.6 (3.3)	20.9 (3.2)	20.3 (3.2)	<0.01
**Ethnicity, % White**	94.9	94.2	95.7	0.34
**SEP, %**				0.53
**Low**	13.4	12.2	15.0	
**Middle**	22.8	23.3	22.0	
**High**	63.8	64.5	63.0	
**Family functioning**	0.00 (−0.68, 0.51)	−0.03 (−0.75, 0.62)	0.01 (−0.61, 0.45)	0.97
**Friendship quality**				
**Good qualities**	0.03 (−0.67, 0.57)	0.02 (−0.7, 0.59)	0.03 (−0.58, 0.53)	0.60
**Difficulties**	−0.04 (−0.49, 0.61)	−0.05 (−0.44, 0.55)	−0.02 (−0.55, 0.65)	0.88
**MVPA, min/d**				
**Weekday**	56.9 (28.8, 98.4)	43.0 (20.7, 72.5)	79.1 (44.0, 122.6)	<0.01
**Weekend**	39.4 (12.0, 85.0)	28.4 (7.2, 61.5)	60.8 (19.7, 114.5)	<0.01
**Sedentary time, hours/d**				
**Weekday**	5.7 (4.1, 7.7)	6.7 (4.7, 8.3)	4.7 (3.6, 6.5)	<0.01
**Weekend**	6.3 (4.7, 8.2)	6.3 (4.8, 8.5)	6.3 (4.6, 8.1)	0.14
**TV viewing, min/d**				
**Weekday**	90 (60, 150)	90 (60, 150)	120 (60, 150)	0.76
**Weekend**	120 (90, 180)	120 (75, 180)	120 (90, 180)	0.76
**Internet use, min/d**				
**Weekday**	60 (30, 120)	90 (45, 120)	60 (30, 120)	<0.01
**Weekend**	90 (60, 180)	120 (60, 180)	60 (30, 120)	<0.01
**Video games, min/d**				
**Weekday**	0 (0, 30)	0 (0, 0)	45 (10, 90)	<0.01
**Weekend**	0 (0, 60)	0 (0, 0)	60 (30, 120)	<0.01
**Homework, min/d**				
**Weekday**	60 (30, 90)	60 (50, 105)	60 (30, 60)	<0.01
**Weekend**	60 (30, 120)	60 (40, 120)	60 (30, 90)	<0.01
**Reading, min/d**				
**Weekday**	20 (0, 30)	30 (0, 45)	10 (0, 30)	<0.01
**Weekend**	20 (0, 60)	30 (0, 60)	5 (0, 60)	<0.01

Data are presented for the subsample providing objective outcome data on weekdays (n = 738). Numbers differ for self-reported sedentary behaviours due to missing data (weekday n = 729, weekend n = 725).

BMI, Body Mass Index; SEP, socioeconomic position; IQR, inter-quartile range; MVPA, moderate to vigorous intensity physical activity; SD, standard deviation.

The associations of family functioning and friendship quality with objectively-measured MVPA and sedentary time are reported in Table 2. In the mutually adjusted model, perceived family functioning was positively associated with weekday MVPA. Participants who scored higher on the good friendship qualities scale accumulated more MVPA on weekdays and weekends and less sedentary time on weekdays.

**Table 2:** **Association of family functioning and friendship quality with physical activity and sedentary time (beta (95%CI))**

	**Family functioning**	**Friendship quality**	
		**Good qualities**	**Difficulties**
**MVPA**			
**Weekday**	0.57 (0.17, 0.98)**	1.13 (0.62, 1.65)**	−0.16 (−0.65, 0.33)
**Weekend**	0.13 (−0.25, 0.51)	0.56 (0.09, 1.02)*	−0.18 (−0.68, 0.33)
**Sedentary time**			
**Weekday**	−2.70 (−7.27, 1.86)	−10.34 (−17.03, −3.66)**	4.09 (−2.56, 10.73)
**Weekend**	−4.74 (−11.17, 1.69)	−3.81 (−12.91, 5.29)	−0.02 (−7.72, 7.68)

Models adjusted for school-level clustering, age, sex, body mass index, ethnicity, sexual maturity, SEP, MFQ and mutually adjusted for family functioning and friendship quality.

95% CI, 95% confidence interval; MVPA, moderate to vigorous intensity physical activity; SEP, socioeconomic position; MFQ, Mood and feelings questionnaire.

Outcome variables (MVPA and sedentary time) were box-cox transformed for analysis.

Weekday n = 738; Weekend n = 677; *p = 0.05; **p = <0.01.

The associations of family functioning and friendship quality with self-reported sedentary behaviours are reported in Table 3 (boys) and Table 4 (girls). Boys from better functioning families were less likely to report playing video games at the weekend or reading for pleasure. Boys who attained higher scores on the good friendship qualities scale were less likely to play video games at the weekend or report high homework on weekdays.

**Table 3:** **Association of family functioning and friendship quality with self-reported sedentary behaviours in boys (OR (95%CI))**

	**Family functioning**	**Friendship quality**	
		**Good qualities**	**Difficulties**
**TV viewing**			
**Weekday**	1.01 (0.71, 1.44)	0.98 (0.66, 1.46)	1.25 (0.87, 1.81)
**Weekend**	0.76 (0.58, 1.00)	0.84 (0.52, 1.35)	1.45 (0.87, 2.43)
**Internet use**			
**Weekday**	1.25 (0.89, 1.77)	0.83 (0.58, 1.18)	0.97 (0.67, 1.41)
**Weekend**	0.96 (0.69, 1.33)	0.83 (0.59, 1.17)	0.91 (0.57, 1.46)
**Video games**			
**Weekday**	0.82 (0.59, 1.13)	1.16 (0.80, 1.69)	0.75 (0.48, 1.15)
**Weekend**	0.73 (0.57, 0.93)*	0.61 (0.44, 0.86)**	1.16 (0.77, 1.75)
**Homework**			
**Weekday**	1.03 (0.67, 1.59)	0.54 (0.31, 0.94)*	1.13 (0.68, 1.87)
**Weekend**	1.07 (0.84, 1.38)	0.81 (0.51, 1.29)	0.94 (0.59, 1.49)
**Reading**			
**Weekday**	0.73 (0.56, 0.96)*	0.77 (0.52, 1.14)	0.82 (0.58, 1.15)
**Weekend**	0.75 (0.58, 0.96)*	0.86 (0.63, 1.18)	0.81 (0.62, 1.05)

Models adjusted for school-level clustering, age, body mass index, ethnicity, sexual maturity, SEP, MFQ and mutually adjusted for family functioning and friendship quality.

OR, odds ratio; 95% CI, 95% confidence interval; SEP, socioeconomic position; MFQ, Mood and feelings questionnaire.

TV viewing, internet use, and homework were dichotomised by median split (low coded 0 (ref); high coded 1).

Playing video games and reading for pleasure were dichotomised as ‘none’ (coded 0 (ref)) versus ‘some’ (coded 1).

Weekday n = 350; weekend n = 346; *p < 0.05; **p < 0.01.

**Table 4:** **Association of family functioning and friendship quality with self-reported sedentary behaviours in girls (OR (95%CI))**

	**Family functioning**	**Friendship quality**	
		**Good qualities**	**Difficulties**
**TV viewing**			
**Weekday**	1.12 (0.90, 1.39)	1.17 (0.81, 1.69)	0.89 (0.65, 1.22)
**Weekend**	1.11 (0.89, 1.40)	1.15 (0.84, 1.57)	0.76 (0.62, 0.93)**
**Internet use**			
**Weekday**	1.11 (0.87, 1.43)	1.15 (0.81, 1.65)	0.97 (0.71, 1.34)
**Weekend**	1.23 (0.94, 1.61)	1.17 (0.88, 1.54)	0.86 (0.71, 1.04)
**Video games**			
**Weekday**	0.83 (0.59, 1.16)	0.76 (0.58, 1.00)*	0.75 (0.52, 1.07)
**Weekend**	0.89 (0.70, 1.13)	1.01 (0.77, 1.33)	0.71 (0.52, 0.97)*
**Homework**			
**Weekday**	0.80 (0.67, 0.94)**	1.13 (0.82, 1.55)	0.70 (0.52, 0.95)*
**Weekend**	1.07 (0.86, 1.32)	1.09 (0.83, 1.43)	0.95 (0.77, 1.17)
**Reading**			
**Weekday**	0.90 (0.71, 1.13)	0.76 (0.52, 1.11)	0.63 (0.49, 0.81)**
**Weekend**	0.86 (0.68, 1.07)	0.61 (0.42, 0.88)**	0.82 (0.56, 1.19)

Models adjusted for school-level clustering, age, body mass index, ethnicity, sexual maturity, SEP, MFQ and mutually adjusted for family functioning and friendship quality.

OR, odds ratio; 95% CI, 95% confidence interval; SEP, socioeconomic position; MFQ, Mood and feelings questionnaire.

TV viewing, internet use, and homework variables were dichotomised by median split (low coded 0 (ref); high coded 1).

Playing video games and reading for pleasure were dichotomised as ‘none’ (coded 0 (ref)) versus ‘some’ (coded 1).

Weekday n = 450; weekend n = 448; *p < 0.05; **p < 0.01.

Amongst girls, better perceived family functioning was associated with reduced odds of high homework on weekdays. A higher score for good friendship qualities was associated with lower odds of girls playing video games during the week or reading for pleasure at the weekend. Girls that reported fewer friendship difficulties had lower odds of high TV viewing or playing video games at the weekend, and lower odds of reading for pleasure or having high homework on weekdays.

## Discussion

This study examined the independent associations of perceived family functioning and friendship quality with physical activity and sedentary behaviours in UK adolescents aged 14 years. The results provide partial support for our hypotheses. In general, adolescents that reported better perceived family functioning, more good friendship qualities or fewer friendship difficulties accumulated more physical activity and reported lower levels of sedentary behaviour. Differential associations were observed for weekdays compared to weekends and there were notable sex differences in the associations between both exposures and self-reported sedentary behaviour. Though confirmation in future research is necessary and careful targeting may be required, findings support the application of strategies directed at improving family functioning and friendship quality within behaviour change interventions.

Adolescents who reported higher levels of family functioning accumulated more objectively measured MVPA on weekdays. This is broadly consistent with previous research which typically has reported that family functioning is positively associated with adolescent health and behaviour, though few studies have examined associations with physical activity [13-15]. In US adolescents aged 14 years, Berge et al. [16] reported that family functioning was positively associated with self-reported physical activity in boys but not girls. We found no evidence of effect modification by sex in our analysis using objectively measured physical activity. The observed association between family functioning and MVPA was limited to weekdays, although the direction of association was similar for weekends. Despite greater available leisure-time at the weekend, adolescents accumulated more MVPA on weekdays compared to weekends as previously reported [36] and consistent with a recent systematic review of between-day differences in young people’s physical activity [37]. Further work to examine whether the characteristics of positive functioning families can be harnessed to promote weekend activity may be beneficial, particularly because it is during the weekend that activity declines most during the transition from childhood to early adolescence [5]. Previous research suggests that parents may be willing to attend physical activity or sedentary behaviour education courses to facilitate behaviour change in their children [38].

Higher scores on the good qualities subscale of our friendship questionnaire were associated with greater MVPA throughout the week and lower sedentary time on weekdays. Few studies have examined the association of friendship quality per se with adolescent physical activity or sedentary behaviour and much of the existing research has relied upon self-report measures of behaviour [17,18,39]. Nonetheless, there is evidence that perceived support, favourable relations with peers and spending more time with friends are positively associated with physical activity in young people [39]. Higher friendship quality may facilitate physical activity through having more friends with shared activity preferences or through the use of active travel modes to visit friends outside of school. Activities such as behavioural hobbies, using active transport and shopping/hanging-out are prevalent in this age group, and may contribute towards the accumulation of MVPA [40]. It is worthwhile to acknowledge the potentially bi-directional nature of this association, as shared sporting experiences, for example, may serve to strengthen and improve friendships between adolescents. Subject to confirmation in future studies, our findings suggest that strategies aimed at developing new or improving existing friendships may be a valuable adjunct to physical activity promotion in the early adolescent population.

A number of significant associations were observed for family functioning and friendship quality with self-reported sedentary behaviours, but associations varied by behaviour, day of week and sex. Despite this variability, the overall trend in the results was that better family functioning and friendship quality was associated with lower levels of sedentary behaviour. Results are broadly consistent with previous research, and a recent systematic review, which has also highlighted the potential influence of family-related constructs on sedentary behaviours in young people and noted that associations may vary across different activities and by sex [11,41-43]. It may be hypothesised that adolescents with good family relations or better quality friendships spend more time with their family or friends, and thus report less time in behaviours that may principally be performed alone, such as playing video games, doing homework or reading. Findings highlight the potential utility of intervention strategies promoting optimal family or friend relations as a means of modifying sedentary behaviours. This may entail, for example, educational sessions (in-person or online) focussed on improving listening and communications skills, developing empathy or providing guidance on dealing with conflict at home or with friends However, careful intervention design is required to ensure that this approach does not direct attention away from academically or developmentally valuable activities, such as homework or reading.

To our knowledge, this is the first study to examine the independent association of family functioning and friendship quality with objectively measured physical activity and sedentary behaviour in early adolescence. Strengths of the current study include the recruitment of a large population-based sample of adolescents and the use of objective methods to assess physical activity and sedentary time. Associations remained after adjustment for depressive symptoms, a potentially significant confounder given the established associations between depressive symptomology, friendship and familial factors and physical activity in this age group [1,44,45]. Analytical models were adjusted for a number of additional potential confounders, though we cannot rule-out the possibility of residual confounding. Due to the cross-sectional design, we are unable to establish causality or temporal sequence of the observed associations. We recognise that there may have been secular changes in sociological factors as well as physical activity and sedentary behaviour since the data were collected. Nonetheless, we maintain that the underlying associations of familial and friendship factors with physical activity and sedentary behaviour are likely to have remained stable. We also acknowledge that all data were not collected concurrently; physical activity and anthropometric measurements were conducted approximately six months subsequent to the demographic and psychosocial assessments. However, the outcome variables are likely to have remained relatively stable over this short period.

## Conclusion

Family functioning and friendship quality exhibit a complex pattern of association with physical activity and sedentary behaviour. Limited previous research exists on the role of these constructs in adolescent’s health behaviour, therefore findings require confirmation in future research. Nonetheless, our findings provide valuable preliminary evidence that strategies targeting family functioning and friendship quality may be a valuable addition to programmes aimed at promoting physical activity or reducing sedentary behaviour in early adolescence.
